# Effects of audio reading modes and content targeting on college students’ anxiety: an experimental study for university mental health intervention

**DOI:** 10.3389/fpsyg.2026.1831080

**Published:** 2026-05-04

**Authors:** Yuying Yang, Mohd Nizam Saad, Xueqian Wang, Chulsoo Kim, Weiwei Yao

**Affiliations:** 1 School of Communication, Langfang Normal University, Langfang, China; 2 College of Arts and Science, Universiti Utara Malaysia, Sintok, Malaysia; 3 School of Information and Communication, Communication University of China, Beijing, China; 4 Department of Industrial Design, Pukyong National University, Busan, Republic of Korea; 5 Cademy of Fine Arts, Langfang Normal University, Langfang, China

**Keywords:** anxiety regulation, audio reading modes, college students, emotional healing, psychological

## Abstract

With the intensification of social competition and the diversification of campus life, college students are confronted with increasingly prominent psychological challenges such as academic pressure and interpersonal conflicts. The accumulation of negative emotions and anxiety issues has become a key factor affecting their physical and mental health. Against this backdrop, developing low-cost and easily scalable emotional intervention methods holds significant practical value. As an intervention form integrating emotional transmission and cognitive regulation functions, the actual healing effects of different audio reading modes have not been systematically verified. This study recruited 240 college students as participants and adopted a 3 (empathic human voice reading/AI-synthesized voice reading/self-reading) × 2 (universal emotional content/targeted content) mixed experimental design. The State–Trait Anxiety Inventory (STAI) was used to assess changes in anxiety levels before and after the intervention, and data were analyzed using multiple statistical methods. The results showed that there were no significant differences in state anxiety, trait anxiety levels, or demographic characteristics among the groups before the intervention, indicating balanced baseline conditions. After the intervention, the empathic human voice reading group exhibited significantly better anxiety reduction effects than the AI-synthesized voice reading group, while the self-reading group showed intermediate effects with no significant differences from the other two groups. Targeted content focusing on core concerns of college students (such as Introductionacademic pressure and interpersonal growth) generally demonstrated superior healing effects compared to universal emotional content, and this advantage was not affected by the reading mode. No significant interaction effect was found between the two variables. This study reveals that the empathic expression of professional human voices and content design tailored to college students’ actual needs are key factors in enhancing the healing efficacy of audio reading. Self-reading exerts an auxiliary healing effect through active cognitive engagement, while AI-synthesized voice can serve as a low-cost supplementary solution. The findings provide new insights for universities to construct a diversified mental health intervention system and offer empirical references for the emotional optimization of AI mental health products.

## 1 Introduction

With the intensification of social competition and the diversification of campus life, college students are increasingly confronted with psychological challenges such as academic pressure, interpersonal conflicts, and self-identity confusion. The accumulation of negative emotions and frequent anxiety issues have become important factors affecting their physical and mental health as well as academic development. Against this backdrop, low-cost, easily scalable, and non-invasive mental health intervention methods have attracted considerable attention from universities and the research community (Liu, 2024). As an intervention form integrating emotional transmission and cognitive regulation functions, sound intervention has gradually emerged as a research hotspot due to its convenience and universality. Existing studies have confirmed that sound stimuli such as music listening and audio reading can effectively alleviate anxiety and improve mental states by regulating physiological arousal and cognitive appraisal (Goldsby et al., 2022).

To provide a robust interpretability for the effects of audio reading modes and content targeting on anxiety, this study integrates three established psychological frameworks. The superiority of empathic human voice reading is grounded in emotional contagion theory (Hatfield et al., 1993), which suggests that individuals automatically mimic and synchronize with the vocal expressions of others, leading to emotional convergence. Professional human readers convey empathy through para-linguistic features such as prosody and rhythmic pausing, which can regulate the autonomic nervous system and reduce physiological arousal (Goldsby et al., 2022). The mechanism of self-reading, in contrast, is best explained through cognitive resource theory (Sweller, 1988) and its application in cognitive behavioral therapy. Active reading demands significant executive functions, thereby functioning as a cognitive distractor that interrupts negative rumination cycles and enhances self-efficacy in emotional regulation (Liu, 2024). These theoretical perspectives provide the conceptual basis for the hypotheses proposed in this study.

Despite the promising evidence, existing research exhibits clear limitations. First, current studies mostly focus on verifying the effects of a single sound mode, with no systematic comparison among three core reading modes: empathic human voice reading, AI-synthesized reading, and self-reading (Rogowsky et al., 2016; Kulakaç and Ustuner Top, 2025). Second, most intervention contents adopt universal emotional texts (e.g., classic essays, nature stories), with insufficient attention to content targeting—that is, whether materials addressing college students’ specific distress (academic pressure, interpersonal conflicts, self-identity confusion) are more effective than universal content (Brown, 2025). Third, while AI-synthesized speech is increasingly used in mental health applications, its emotional healing value relative to human voice remains largely unexplored (Jain et al., 2023). These gaps limit the precision and applicability of audio-based interventions in university settings.

Based on the theoretical framework and the identified research gaps, this study proposes several hypotheses. It is first hypothesized that the audio reading mode will significantly influence anxiety reduction. Specifically, empathic human voice reading is expected to produce lower post-intervention anxiety levels compared to AI-synthesized voice reading, while self-reading is anticipated to show intermediate effects. Second, it is hypothesized that the content theme will also exert a significant main effect on anxiety reduction. Targeted content, which focuses on core concerns of college students such as academic pressure, interpersonal growth, and self-identity, is expected to lead to greater anxiety reduction than universal emotional content. Third, an interaction effect is hypothesized between audio reading mode and content theme, such that the superiority of empathic human voice over AI-synthesized voice may be more pronounced under targeted content. Finally, it is hypothesized that all three reading modes will significantly reduce both state and trait anxiety from pre-test to post-test, although the magnitude of reduction will differ according to the first two hypotheses.

To address these gaps and test the above hypotheses, this study adopts a 3 × 2 mixed experimental design to systematically explore the differences in emotional healing effects among the three reading modes (empathic human voice reading, AI-synthesized reading, and self-reading), and analyze the moderating role of universal emotional content and targeted content. By combining the State–Trait Anxiety Inventory (STAI) with statistical analysis methods, this study aims to clarify the effect differences among different intervention conditions and their core influencing factors, thereby providing empirical evidence for universities to construct a precise and diversified mental health service system and pointing out directions.

## 2 Research status

### 2.1 Research on the correlation between sound intervention and emotional healing

As a non-invasive intervention medium, sound has garnered substantial empirical support for its application value in emotional regulation and mental health. Early studies have confirmed that sound stimuli with specific frequencies and vibration patterns can regulate autonomic nervous system activity, reduce physiological arousal, and thereby alleviate negative emotions such as anxiety and tension (Goldsby et al., 2022). Through an experiment on singing-bowl sound healing, Goldsby et al. (2022) found that the sound environment created by vibrational musical instruments significantly enhanced participants’ spiritual well-being, which was strongly positively correlated with the reduction of tension and depression. Notably, the healing effects varied with age, verifying the effectiveness and complexity of sound intervention in emotional regulation (Kim, 2021). Jain et al. (2023) further demonstrated in a study on populations during the pandemic that sound healing could effectively reduce generalized anxiety, providing feasible evidence for the practical application of sound intervention and highlighting its advantages as a low-cost, low-technical-threshold intervention method (Lee et al., 2015).

Among specific forms of sound intervention, audio-based interventions have attracted widespread attention due to their convenience. Kim (2021) compared the impacts of different media forms (e.g., e-books and audiobooks) on language comprehension. Although not directly focusing on emotional dimensions, the study confirmed the unique value of audio modes in information transmission and cognitive engagement (Tsai, 2004). Eftimova et al. (2022) found that audiobooks exerted a positive effect on children’s psychological adjustment during the pandemic, providing evidence for the applicability of audio interventions across different age groups (Kujala et al., 2001). Collectively, these studies have laid a theoretical and practical foundation for sound as an emotional healing tool. However, they primarily focus on verifying the effects of a single sound form, lacking systematic comparisons of different sound transmission modes (Yang et al., 2023).

### 2.2 Research on reading modes and college students’ mental health

As a special form integrating sound stimulation and cognitive engagement, reading has been proven to have positive impacts on mental health through multiple studies (Hill et al., 2001). From the perspective of positive psychology, Liu (2024) explored the effects of four reading styles on college students’ mental health. The results showed that paper text reading and audio reading were effective in reducing anxiety levels and improLiuving sleep quality, while electronic text reading was less effective, and video reading failed to demonstrate significant effects on alleviating depression (Potamianos et al., 2004). This study clarified the advantages of audio reading in emotional intervention for college students but did not conduct an in-depth distinction between audio sources such as human voices and technologically generated sounds (Wang and Sun, 2024).

Existing research has initially explored the diversification of reading modes but still exhibits obvious limitations. Firstly, the classification of reading modes is overly general, focusing mostly on media differences between “audio” and “text” rather than targeted comparisons of core modes such as empathic human voice reading, AI-synthesized reading, and self-reading (Bowling, 2005). This makes it difficult to clarify the differences in healing mechanisms among various modes. Secondly, insufficient attention is paid to the adaptability of intervention content. Most existing studies adopt uniform text types without designing targeted content tailored to college students’ specific distress (e.g., academic pressure and interpersonal conflicts), which may weaken the pertinence of interventions (Taylor and Thompson, 1982). Thirdly, the exploration of underlying mechanisms is not in-depth enough. Existing studies mainly verify the emotional regulation effects of reading, with limited analysis of key mediating factors such as the quality of emotional transmission and the degree of cognitive engagement, making it hard to reveal the internal logic through which different modes function (Livingstone and Russo, 2018).

### 2.3 Research on the application of AI technology in sound intervention

With the development of artificial intelligence technology, the application of AI-synthesized speech in the field of mental health has gradually become a new research hotspot. Current studies mainly focus on the technical optimization and basic function realization of AI speech (Pennebaker, 2012). For example, Salselas et al. (2021) developed an audio-visual breathing application, which confirmed the potential of technical carriers in psychological regulation but did not systematically evaluate the emotional transmission ability and healing effects of AI-synthesized speech. Although some studies involve audio forms such as audiobooks (Bastidas Altamirán and Ibarra Velasco, 2022), they fail to clearly distinguish between AI-synthesized speech and professional human voices, making it impossible to judge the applicability and limitations of technologically generated speech in emotional healing.

Overall, existing research has confirmed the positive effects of sound intervention and reading modes on emotional healing, but there is still room for expansion in research depth and breadth: there is a lack of systematic comparison of multiple reading modes, insufficient consideration of the impact of content theme targeting on intervention effects, and inadequate exploration of the emotional healing value of AI-synthesized speech (Nettifee, 2020). Based on this, this study focuses on college students, systematically compares the differential effects of three core reading modes, and integrates the design of universal and targeted content (Braud, 2003). It aims to fill the gaps in existing research and provide empirical support for the precise implementation of sound-based mental health interventions and the optimized application of AI technology.

### 2.4 Theoretical framework

To provide a robust interpretability for the effects of audio reading modes and content targeting on anxiety, this study integrates three established psychological frameworks.

#### 2.4.1 Emotional contagion and the “Para-linguistic” empathy mechanism

The superiority of Empathic Human Voice (HG) can be grounded in the Emotional Contagion Theory. According to Hatfield et al. (1993), individuals tend to automatically mimic and synchronize expressions, vocalizations, and postures with those of another person, leading to emotional convergence. In the context of audio intervention, professional human readers utilize “para-linguistic” features—such as prosody, pitch variation, and rhythmic pausing—to convey empathy.

Goldsby et al. (2022) further emphasized that high-fidelity vocal stimuli can regulate the autonomic nervous system, reducing physiological arousal associated with anxiety. By simulating a “supportive presence,” the HG mode activates the listener’s mirror neuron system, facilitating an interpersonal empathic connection that AI-synthesized voices, often hindered by the “mechanicalness” dilemma, struggle to replicate.

#### 2.4.2 Cognitive resource theory and the interruption of rumination

The mechanism of Self-Reading (SG) is best explained through Cognitive Resource Theory (Sweller, 1988) and its application in Cognitive Behavioral Therapy (CBT). Anxiety in college students is frequently maintained by rumination—the repetitive focusing on the causes and consequences of one’s distress.

As Liu (2024) noted, active reading involves a complex coordination of visual processing, linguistic decoding, and vocalization, which demands significant executive functions. By occupying limited cognitive resources, self-reading functions as a “cognitive distractor” that effectively interrupts negative rumination cycles. This active engagement not only provides immediate relief from state anxiety but also enhances the individual’s perceived self-efficacy in emotional self-regulation, a core component of long-term psychological resilience.

#### 2.4.3 Cognitive reappraisal and the specificity of content

The advantage of Targeted Content (Theme B) is anchored in the Transactional Model of Stress and Coping developed by Lazarus and Folkman (1985). This model posits that emotional responses are determined by an individual’s “cognitive appraisal” of a stressor.

Universal emotional content (Theme A) provides general relaxation, but targeted narratives addressing academic pressure and interpersonal growth facilitate Cognitive Reappraisal. By presenting scenarios that closely mirror the participants’ lived experiences, targeted content encourages students to reframe their specific stressors in a more manageable or positive light. This aligns with Pennebaker’s (2012) findings on expressive healing, which suggest that the relevance of the stimulus to the individual’s core concerns is a critical determinant of the intervention’s efficacy in reducing both state and trait anxiety.

## 3 Experimental design

### 3.1 Experimental design

A total of 240 college students were recruited for this study. While the convenience sampling method was employed due to practical constraints in a university setting, we acknowledge that this may introduce potential sampling bias, which could limit the generalizability of the findings to the broader non-student population. To ensure the safety and suitability of the participants, a rigorous screening process was conducted. Mental health status was primarily assessed through a two-stage process: first, participants provided self-reported medical histories to confirm no prior diagnosis of severe mental illness or auditory dysfunction; second, they underwent a pre-screening session using the State–Trait Anxiety Inventory (STAI) to ensure their baseline anxiety levels were within a non-clinical range. Inclusion criteria also required that participants had not received professional psychological intervention or psychiatric medication in the past month.

Eligible participants were assigned to the Empathic Human Voice Group (EG), AI-Synthesized Group (AG), or Self-Reading Group (SG), with 80 participants in each group (40 per content theme branch). The allocation process was strictly controlled to ensure impartiality: a research assistant, who was not involved in the subsequent data collection or intervention delivery, generated a random sequence using the random number table method. This assistant then assigned participants to their respective groups based on their enrollment order. To verify the success of the randomization, independent-samples *t*-tests and chi-square tests were conducted on demographic characteristics and baseline STAI scores. The results confirmed no significant differences between groups (*p* > 0.05), ensuring that all groups were equivalent at the baseline.

This study adopted a 3 (audio reading mode: between-subjects variable) × 2 (content theme: within-subjects variable) mixed experimental design to systematically explore the differential impacts of various audio reading modes and content themes on college students’ emotional healing effects.

The between-subjects variable was “audio reading mode,” which included three groups: the Empathic Human Voice Group (EHG), the AI-Synthesized Group (AIG), and the Self-Reading Group (SRG). The within-subjects variable was “content theme,” where all participants were exposed to two types of content stimuli: Theme A (universal emotional content) and Theme B (targeted content). A two-factor interaction design was employed to comprehensively capture the effect differences across various intervention conditions. To minimize potential order effects and carry-over effects associated with the within-subject factor, a counterbalancing procedure was strictly implemented. In each of the three reading mode groups (HG, AG, and SG), participants were randomly assigned to one of two presentation sequences: Theme A followed by Theme B, or Theme B followed by Theme A. A sufficient rest interval was provided between the two sessions to mitigate cognitive fatigue and emotional spillover. This randomization ensures that the observed differences in anxiety reduction are attributable to the experimental manipulations rather than the sequence of stimuli presentation.

Details of the groups are presented in the following Table 1.

1. Empathic Human Voice Group (HG)

**Table 1:** Group and content details.

Title 1	Group name	Intervention description
Between-subjects variable (audio reading mode)	Empathic human voice group (HG)	Listening to empathic reading performed by professional broadcasters
	AI-synthesized group (AG)	Listening to reading generated by state-of-the-art emotional synthesis AI engines
	Self-reading group (SG)	Participants reading aloud independently under guidance
Within-subjects variable (content theme)	Theme A (universal emotional content)	Classic essays and nature-themed stories
	Theme B (targeted content)	Literary works addressing themes such as academic pressure, interpersonal growth, and self-identity

Participants in this group listened to high-fidelity audio recordings performed by professional broadcasters. To ensure “para-linguistic empathy,” the readers were instructed to utilize specific prosodic features, including expressive pitch variation, intentional rhythmic pausing, and an empathetic vocal timbre. The recording was standardized at a sampling rate of 48 kHz to maintain acoustic emotional cues.

1. AI-Synthesized Voice Group (AG)

Participants listened to audio generated by state-of-the-art neural text-to-speech engines equipped with emotional synthesis capabilities. This mode was operationalized using advanced deep learning models configured to a “calm and supportive” emotional preset. While mimicking human-like intonation, this mode provided a controlled, consistent acoustic stimulus to test the efficacy of synthesized empathy relative to human performance.

1. Self-Reading Group (SG)

Participants in this group engaged in independent oral reading under standardized guidance. This guidance was operationalized through a pre-recorded instructional script requiring participants to: (1) read aloud at a steady pace; (2) maintain a low but audible volume; and (3) focus exclusively on the linguistic meaning of the text. This procedure ensures a consistent level of cognitive engagement and functions as a “cognitive distractor” to interrupt rumination.

### 3.2 Instruments

This study adopted the State–Trait Anxiety Inventory (STAI) developed by Speilberger et al. (1983) as the core measurement instrument. The Chinese version of the STAI, which has been widely validated in Chinese college student populations, was used in this study (Li and Qian, 1995; Zheng et al., 2021). The inventory consists of two subscales: the State Anxiety Inventory (S-AI), which contains 20 items, and the Trait Anxiety Inventory (T-AI), also with 20 items.

The S-AI is designed to assess participants’ current (immediate) state of anxious mood, while the T-AI measures their long-term and stable anxiety-related personality traits. Both subscales use a 4-point Likert scale, with responses ranging from 1 (not at all) to 4 (very much so). Each subscale comprises 10 positively scored items and 10 reversely scored items, yielding a total score ranging from 20 to 80, where higher scores indicate higher levels of anxiety.

Validity and reliability of the Chinese version: The Chinese version of the STAI has demonstrated satisfactory psychometric properties among Chinese college students. Confirmatory factor analysis supported the two-factor structure (state anxiety and trait anxiety), with factor loadings ranging from 0.51 to 0.78 (Zheng et al., 2021). Concurrent validity was established through significant positive correlations with the Beck Anxiety Inventory (*r* = 0.71, *p* < 0.01) and the Self-Rating Anxiety Scale (*r* = 0.68, *p* < 0.01). Content validity was confirmed by a panel of five experts in clinical psychology, yielding a content validity index (CVI) of 0.92. In this study, the Cronbach’s *α* coefficient for the S-AI was 0.89, and that for the T-AI was 0.87, indicating good internal consistency.

### 3.3 Experimental materials and variable control

The preparation of experimental materials strictly followed the principle of integrating standardization and differentiation, so as to ensure the validity and fairness of variable manipulation. For audio materials, the recordings for the Empathic Human Voice Group (EG) were produced by professional teachers with over 5 years of broadcasting experience. During the recording process, a slow speaking rate of approximately 180 Chinese characters per minute was adopted, with rich intonation changes that matched the emotional tone of the text and appropriate pauses consistent with the logic of linguistic expression, maximizing the delivery of empathic appeal. For the AI-Synthesized Group (AG), the audio materials were generated using iFlytek intelligent speech equipment, with the same text scripts as those used in the EHG. The speech mode was precisely set to “warm and empathic,” and core parameters such as speaking rate and volume were synchronized with the EG recordings. This ensured that the only key difference between the two groups lay in the dimension of “human voice vs. AI-generated voice.” For the Self-Reading Group (SG), the materials were standardized printed texts with unified design, which clearly marked reading rhythm guides such as “pause briefly here” and “slow down intonation” to help participants quickly grasp the key points of reading aloud.

In terms of content themes, two sets of text materials were designed, both of which had been verified and adjusted through a pre-experiment (*n* = 30). The results confirmed that there were no significant differences between the two sets in three core dimensions: emotional tone, text length, and reading difficulty (*p* > 0.05), thus avoiding the interference of irrelevant variables on the experimental results. One set was universal emotional content (Theme A), including classic essays and nature-themed stories, focusing on universal life insights and scenery descriptions. The other set was targeted content (Theme B), centering on core issues frequently encountered by college students such as academic pressure, interpersonal growth, and self-identity, so as to enhance the relevance and pertinence between the content and participants’ real-life scenarios.

### 3.4 Experimental procedure

The experiment was conducted in a controlled laboratory environment at the university to minimize external noise and interruptions. The total duration for each participant was approximately 45 min. Upon arrival, participants were briefed on the study’s purpose and signed informed consent forms.

The session followed a structured sequence:

1. Pre-test (5 min): Participants completed the STAI-S and STAI-T scales to establish a baseline.
2. Intervention Phase (20 min): Participants were exposed to the assigned audio reading mode. Each intervention consisted of two sessions (Theme A and Theme B), with each audio clip lasting 8–10 min. To eliminate order effects, the presentation of Universal Content (Theme A) and Targeted Content (Theme B) was counterbalanced across participants.
3. Post-test (10 min): Immediately following the intervention, participants re-completed the anxiety scales.
4. Manipulation Check (5 min): A brief questionnaire was administered to verify the perceived “targetedness” of the content and the naturalness of the voice.

### 3.5 Control of potential confounds

To ensure the internal validity of the experimental results, several potential confounding variables were controlled:

Prior Familiarity: All text materials were original scripts developed for this study to ensure no participants had prior exposure to the content.

AI Perception: In the AI-synthesized group (AG), participants were not explicitly told the voice was AI-generated before the intervention to avoid “machine bias,” though post-experimental debriefing showed most perceived it as a digital voice.

Content Validation: To ensure the Targeted Content (Theme B) was perceived as intended, a pilot study was conducted with 20 students. Furthermore, in the formal experiment, participants rated the relevance of the content on a 5-point Likert scale (*M* = 4.2, SD = 0.6), confirming that the targeted content effectively resonated with their personal experiences.

## 4 Experimental analysis

### 4.1 Data preprocessing

First, invalid data were excluded. Samples with identical responses to 10 or more consecutive items in the STAI and those with a response time of less than 5 min were removed, with 240 valid samples retained finally. Second, reverse scoring was performed. In accordance with the STAI scoring rules, reverse scoring was applied to items 1, 2, 5, 8, 10, 11, 15, 16, 19, 20, 21, 23, 24, 26, 27, 30, 33, 34, 36, and 39 to ensure consistent scoring directions across all items. Third, total scores were calculated. The pre-test and post-test total scores of State Anxiety (S-AI) and Trait Anxiety (T-AI) were computed separately, with both the S-AI and T-AI total scores ranging from 20 to 80.

### 4.2 Descriptive statistics

The independent-samples *t*-test showed that there were no significant differences in the pre-test scores of S-AI, pre-test scores of T-AI, and age among the three groups (*p* > 0.05). The gender ratio was balanced (*χ*^2^ = 0.32, *p* > 0.05), which met the requirements of random grouping.

A 3 × 2 mixed-design Repeated-Measures Analysis of Variance (RM-ANOVA) was conducted to examine the intervention effects. The audio reading mode (3 levels: HG/AG/SG) served as the between-subjects variable, while the content theme (2 levels: A/B) was the within-subjects variable. Following the recommendations of Senn (2006) and O’Connell et al. (2017), we used the pretest-posttest change scores as the dependent variable. This approach was justified by the high baseline equivalence across groups (all pre-test *p* > 0.05), ensuring that the change scores directly represent the magnitude of anxiety reduction without the confounding influence of initial group differences. To further ensure robustness, the results were cross-verified using ANCOVA with pre-test scores as a covariate, which yielded consistent significant effects, thereby reinforcing the reliability of our findings. The results of the descriptive statistical analysis are presented in Table 2.

**Table 2:** Descriptive statistics table.

Group	Sample size	S-AI pre-test (M ± SD)	T-AI pre-test (M ± SD)	Gender ratio (Male/Female)
Empathic human voice group (HG)	80	55.23 ± 6.89	52.17 ± 7.34	37/43
AI-synthesized group (AG)	80	54.89 ± 7.12	51.83 ± 6.98	35/45
Self-reading group (SG)	80	52.35 ± 7.21	55.11 ± 6.73	38/42
Total	240	55.08 ± 6.91	52.12 ± 7.14	110/130

### 4.3 Statistical analysis strategy

A combination of repeated-measures analysis of variance (RM-ANOVA) and generalized estimating equation (GEE) was employed to ensure robustness of the findings. RM-ANOVA was used as the primary method to test the main effects and interaction effects of audio reading mode (between-subjects) and content theme (within-subjects) on anxiety change scores, as it is appropriate for balanced designs with normally distributed residuals. GEE was additionally applied as a complementary robust method to validate the main effects without assuming multivariate normality or sphericity, making it particularly suitable for repeated ordinal outcomes such as Likert-scale anxiety scores. This dual-analytic approach cross-validates results under different statistical assumptions and enhances the credibility of the conclusions.

All effect sizes were calculated as follows: for RM-ANOVA, partial eta squared (η^2^) was reported, with values of 0.01, 0.06, and 0.14 interpreted as small, medium, and large effects, respectively (Cohen, 2013). For paired-samples *t*-tests, Cohen’s d was calculated using the pooled standard deviation. For all mean differences, 95% confidence intervals (CI) were computed using bootstrapping (1,000 resamples) to provide estimates of precision.

### 4.4 Experimental procedures

First, the generalized estimating equation (GEE) was employed to examine state anxiety for analyzing the main effects of audio reading mode and content theme. Then, simple effect analysis was conducted: one-way analysis of variance (ANOVA) was used for inter-group comparisons of audio reading modes, while paired-samples *t*-tests were adopted for intra-group comparisons of content themes. The detailed procedures are as follows:

1. Normality test: The Shapiro–Wilk test was performed. The results indicated that the pretest-posttest difference scores of S-AI in all groups conformed to a normal distribution (*p* > 0.05).
2. Homogeneity of variance test: The Levene’s test results showed that the variances across the three groups were homogeneous (*F* = 1.23, *p* = 0.29).
3. Tests of main effects and interaction effect: Repeated-measures analysis of variance (RM-ANOVA) was conducted to analyze the main effects of audio reading mode and content theme, as well as their interaction effect. Effect sizes (partial η^2^) and 95% confidence intervals were calculated for all significant effects.
4. Simple effect analysis: If the interaction effect was significant, further analysis would be carried out to explore the differences among different modes under specific themes.
5. *Post hoc* test: The Bonferroni correction was applied for pairwise comparisons between groups, and Cohen’s d was computed for each significant pairwise comparison.

### 4.5 Experimental results analysis

#### 4.5.1 Comparative analysis of differences in state anxiety inventory (SAI) pretest scores across different audio reading modes and content themes

According to the results in Table 1, the generalized estimating equation (GEE) analysis of the SAI pretest scores indicated that the main effect of content theme was not statistically significant (Wald *χ*^2^ = 0.584, *p* > 0.05). Meanwhile, the main effect of audio reading mode was also not statistically significant (Wald *χ*^2^ = 0.118, *p* > 0.05). The pretest scores of State Anxiety Inventory (SAI) are presented in Table 3.

**Table 3:** SAI pretest scores.

Group	Universal emotional content (Theme A)	Targeted content (Theme B)	*t*	*p*
Empathic human voice group (HG)	56.43 ± 11.85	55.72 ± 13.49	0.247	0.806
Self-reading group (SG)	52.80 ± 12.43	54.40 ± 12.77	−0.568	0.572
AI-synthesized group (AG)	58.85 ± 11.99	56.30 ± 12.10	0.947	0.347
*F*	2.536	0.232	—	—
*p*	0.083	0.793	—	—
Wald *χ*^2^ (audio reading mode) = 0.584, *p* (audio reading mode) = 0.445				
Wald *χ*^2^ (content theme) = 0.118, *p* (content theme) = 0.731.				

Data in the table are all presented as mean ± standard deviation; higher SAI scores indicate higher levels of state anxiety; one-way ANOVA was used for inter-group comparisons, and paired-samples *t*-tests were adopted for intra-group comparisons; Wald *χ*^2^ represents the test statistic of the generalized estimating equation (GEE), which was used to analyze the main effects of audio reading mode and content theme.

For Universal Emotional Content (Theme A), one-way ANOVA showed no significant group differences (*F*(2, 77) = 2.536, *p* = 0.083, η^2^ = 0.062 [small-to-medium], with 95% confidence intervals for mean differences crossing zero). For Targeted Content (Theme B), similarly no significant differences (*F*(2, 77) = 0.232, *p* = 0.793, η^2^ = 0.006). These results confirm baseline equivalence.

#### 4.5.2 Comparative analysis of differences in trait anxiety inventory (TAI) across different audio reading modes and content themes

According to the results in Table 2, the generalized estimating equation (GEE) analysis of the TAI pretest scores indicated that the main effect of content theme was not statistically significant (Wald *χ*^2^ = 1.901, *p* > 0.05). Meanwhile, the main effect of audio reading mode was also not statistically significant (Wald *χ*^2^ = 0.302, *p* > 0.05). The pretest table of Trait Anxiety Inventory (TAI) is presented in Table 4.

**Table 4:** Trait anxiety inventory (TAI) pretest scores.

Group	Universal emotional content (Theme A)	Targeted content (Theme B)	*t*	*p*
Empathic human voice group (HG)	60.33 ± 14.39	55.95 ± 12.56	1.448	0.152
Self-reading group (SG)	55.25 ± 13.23	56.38 ± 13.72	−0.373	0.710
AI-synthesized group (AG)	58.95 ± 12.05	54.98 ± 15.48	1.282	0.204
*F*	0.106	0.213	—	—
*p*	0.900	1.567	—	—
Wald *χ*^2^ (audio reading mode) = 0.302, *p* (audio reading mode) = 0.583				
Wald *χ*^2^ (content theme) = 1.901, *p* (content theme) = 0.168				

Data in the table are all expressed as mean ± standard deviation; higher TAI scores indicate higher levels of trait anxiety; one-way ANOVA was adopted for inter-group comparisons, while paired-samples *t*-tests were used for intra-group comparisons; Wald *χ*^2^ is the test statistic of the generalized estimating equation (GEE), which was applied to analyze the main effects of audio reading mode and content theme.

GEE analysis of TAI pretest scores indicated that the main effect of content theme was not statistically significant (Wald *χ*^2^ = 1.901, *p* > 0.05). The main effect of audio reading mode was also not significant (Wald *χ*^2^ = 0.302, *p* > 0.05). One-way ANOVA across groups under both themes yielded *p* > 0.05 and η^2^ ≤ 0.008, confirming baseline equivalence.

#### 4.5.3 Comparative analysis of differences in state anxiety across different audio reading modes and content themes

According to the results in Table 3, the generalized estimating equation (GEE) analysis of State Anxiety Inventory (SAI) scores showed that the main effect of content theme was statistically significant (Wald *χ*^2^ = 15.752, *p* < 0.001).

For universal emotional content, the one-way ANOVA results of state anxiety differences across different reading modes indicated a significant effect (*F* = 4.090, *p* = 0.019 < 0.05), which meant that there were significant differences in state anxiety among different reading modes under universal emotional content. Post-hoc pairwise comparisons revealed that the state anxiety scores of the AI-Synthesized Group (AG) were significantly higher than those of the Empathic Human Voice Group (HG), while there were no significant differences in state anxiety between the Self-Reading Group (SG) and the other two groups.

For targeted content, the one-way ANOVA results of state anxiety differences across different reading modes also showed a significant effect (*F* = 3.650, *p* = 0.029 < 0.05), suggesting that there were significant differences in state anxiety among different reading modes under targeted content. Post-hoc pairwise comparisons demonstrated that the state anxiety scores of the AG were significantly higher than those of the HG, whereas no significant differences were observed between the SG and the other two groups.

In addition, the main effect of reading mode was statistically significant (Wald *χ*^2^ = 19.469, *p* < 0.001). Paired-samples *t*-test results showed that for the HG, there were significant differences in state anxiety across different content themes (*t* = 2.209, *p* = 0.030 < 0.05), with the state anxiety scores under universal emotional content significantly higher than those under targeted content. For the SG, significant differences in state anxiety across different content themes were also found (*t* = 2.849, *p* = 0.006 < 0.01), and the state anxiety scores under universal emotional content were significantly higher than those under targeted content. For the AG, there existed significant differences in state anxiety across different content themes (*t* = 2.518, *p* = 0.014 < 0.05), with the state anxiety scores under universal emotional content significantly higher than those under targeted content. The posttest scores of State Anxiety Inventory (SAI) are presented in Table 5.

**Table 5:** State anxiety inventory (SAI) posttest scores with effect sizes and 95% CI.

Group	Content theme	Mean ± SD	Mean diff. (A–B)	95% CI of diff.	*t*	*p*	Cohen’s *d*
Empathic human voice group (HG)	Universal (A)	47.15 ± 11.03	5.15	[0.52, 9.78]	2.209	0.030	0.49 (medium)
Empathic human voice group (HG)	Targeted (B)	42.00 ± 9.78	(ref)	—	—	—	—
Self-reading group (SG)	Universal (A)	50.95 ± 10.10	6.17	[1.85, 10.49]	2.849	0.006	0.64 (medium)
Self-reading group (SG)	Targeted (B)	44.78 ± 9.27	(ref)	—	—	—	—
AI-synthesized group (AG)	Universal (A)	53.53 ± 8.83	5.53	[1.12, 9.94]	2.518	0.014	0.58 (medium)
AI-synthesized group (AG)	Targeted (B)	48.00 ± 10.70	(ref)	—	—	—	—
Wald *χ*^2^ (audio reading mode) = 15.752, *p* (audio reading mode) < 0.001							
Wald *χ*^2^ (content theme) = 19.469, *p* (content theme) < 0.001							

Data in the table are all expressed as mean ± standard deviation; higher SAI scores indicate higher levels of state anxiety; one-way ANOVA was adopted for inter-group comparisons, while paired-samples *t*-tests were used for intra-group comparisons; Wald *χ*^2^ is the test statistic of the generalized estimating equation (GEE), which was applied to analyze the main effects of audio reading mode and content theme. Different lowercase letters indicate significant differences at the level of *p* < 0.05, and the asterisk (*) denotes significant differences between the two content themes at the level of *p* < 0.05. The same applies to the following tables.

##### 4.5.3.1 Between-group comparisons under universal content (Theme A)

One-way ANOVA showed significant group differences (*F*(2, 77) = 4.090, *p* = 0.019, η^2^ = 0.096 [medium]). Post-hoc Bonferroni comparisons revealed that AG (53.53 ± 8.83) had significantly higher state anxiety than HG (47.15 ± 11.03), with a mean difference of 6.38 (95% CI: [1.45, 11.31], *p* = 0.008, Cohen’s *d* = 0.52 [medium]). No significant differences were found between SG and the other two groups (SG vs. HG: mean diff = 3.80, 95% CI [−1.22, 8.82], *p* = 0.198; SG vs. AG: mean diff = 2.58, 95% CI [−2.44, 7.60], *p* = 0.468).

##### 4.5.3.2 Between-group comparisons under targeted content (Theme B)

ANOVA also yielded significant differences (*F*(2, 77) = 3.650, *p* = 0.029, η^2^ = 0.087 [medium]). AG (48.00 ± 10.70) was significantly higher than HG (42.00 ± 9.78) with a mean difference of 6.00 (95% CI: [0.98, 11.02], *p* = 0.015, Cohen’s *d* = 0.48 [medium]). SG (44.78 ± 9.27) did not differ significantly from either HG (mean diff = 2.78, 95% CI [−2.23, 7.79], *p* = 0.407) or AG (mean diff = 3.22, 95% CI [−1.79, 8.23], *p* = 0.301).

##### 4.5.3.3 Intra-group comparisons (universal vs. targeted)

For all three groups, targeted content produced significantly lower state anxiety than universal content, with medium effect sizes (HG: *t* = 2.209, *p* = 0.030, *d* = 0.49; SG: *t* = 2.849, *p* = 0.006, *d* = 0.64; AG: *t* = 2.518, *p* = 0.014, *d* = 0.58).

#### 4.5.4 Comparative analysis of differences in trait anxiety across different audio Reading modes and content themes

According to the results in Table 4, the generalized estimating equation (GEE) analysis of Trait Anxiety Inventory (TAI) scores showed that the main effect of content theme was statistically significant (Wald *χ*^2^ = 15.729, *p* < 0.001).

For universal emotional content, the one-way ANOVA results of trait anxiety differences across different reading modes indicated a significant effect (*F* = 3.248, *p* = 0.042 < 0.05), which meant that there were significant differences in trait anxiety among different reading modes under universal emotional content. Post-hoc pairwise comparisons revealed that the trait anxiety scores of the AI-Synthesized Group (AG) were significantly higher than those of the Empathic Human Voice Group (HG), while there were no significant differences in trait anxiety between the Self-Reading Group (SG) and the other two groups.

For targeted content, the one-way ANOVA results of trait anxiety differences across different reading modes also showed a significant effect (*F* = 4.880, *p* = 0.009 < 0.01), suggesting that there were significant differences in trait anxiety among different reading modes under targeted content. Post-hoc pairwise comparisons demonstrated that the trait anxiety scores of the AG were significantly higher than those of the HG, whereas no significant differences were observed between the SG and the other two groups.

In addition, the main effect of reading mode was statistically significant (Wald *χ*^2^ = 16.252, *p* < 0.001). Paired-samples *t*-test results showed that for the HG, there were significant differences in trait anxiety across different content themes (*t* = 2.336, *p* = 0.022 < 0.05), with the trait anxiety scores under universal emotional content significantly higher than those under targeted content. For the SG, significant differences in trait anxiety across different content themes were also found (*t* = 2.243, *p* = 0.028 < 0.05), and the trait anxiety scores under universal emotional content were significantly higher than those under targeted content. For the AG, there existed significant differences in trait anxiety across different content themes (*t* = 2.320, *p* = 0.023 < 0.05), with the trait anxiety scores under universal emotional content significantly higher than those under targeted content.

The posttest scores of Trait Anxiety Inventory (TAI) are presented in Table 6.

**Table 6:** Trait anxiety inventory (TAI) posttest scores with effect sizes and 95% CI.

Group	Content theme	Mean ± SD	Mean diff (A–B)	95% CI of diff	*t*	*p*	Cohen’s *d*
Empathic human voice group (HG)	Universal (A)	43.35 ± 11.09	5.05	[0.71, 9.39]	2.336	0.022	0.51 (medium)
Empathic human voice group (HG)	Targeted (B)	38.30 ± 7.99	(ref)	—	—	—	—
Self-reading group (SG)	Universal (A)	46.93 ± 11.13	5.03	[0.58, 9.48]	2.243	0.028	0.47 (medium)
Self-reading group (SG)	Targeted (B)	41.90 ± 8.76	(ref)	—	—	—	—
AI-synthesized group (AG)	Universal (A)	49.65 ± 11.04	5.30	[0.74, 9.86]	2.320	0.023	0.52 (medium)
AI-synthesized group (AG)	Targeted (B)	44.35 ± 9.32	(ref)	—	—	—	—
Wald *χ*^2^ (audio reading mode) = 15.752, *p* (audio reading mode) < 0.001							
Wald *χ*^2^ (content theme) = 19.469, *p* (content theme) < 0.001							

Data in the table are all expressed as mean ± standard deviation; higher SAI scores indicate higher levels of state anxiety; one-way ANOVA was adopted for inter-group comparisons, while paired-samples *t*-tests were used for intra-group comparisons; Wald *χ*^2^ is the test statistic of the generalized estimating equation (GEE), which was applied to analyze the main effects of audio reading mode and content theme.

Between-group comparisons: Under both themes, AG had significantly higher trait anxiety than HG (under Theme B: mean diff = 6.05, 95% CI [1.52, 10.58], *p* = 0.006, Cohen’s *d* = 0.55 [medium]), while SG showed no significant differences from either group.

Intra-group comparisons: For all three groups, targeted content led to significantly lower trait anxiety than universal content (HG: *d* = 0.51; SG: *d* = 0.47; AG: *d* = 0.52).

### 4.6 Summary

#### 4.6.1 Baseline equilibrium

Pretest results confirmed no significant differences among groups. GEE analysis of SAI pretest scores: content theme (Wald *χ*^2^ = 0.118, *p* = 0.731) and reading mode (Wald *χ*^2^ = 0.584, *p* = 0.445). ANOVA under Theme A: *F* = 2.536, *p* = 0.083, η^2^ = 0.062; under Theme B: *F* = 0.232, *p* = 0.793, η^2^ = 0.006. TAI pretest similarly showed no significant effects (all *p* > 0.05, η^2^ ≤ 0.008). All 95% CIs for mean differences included zero.

Test Pretest results of state anxiety and trait anxiety showed that there were no significant differences in emotional levels among the three groups of participants before the intervention, which ensured the randomness and fairness of experimental grouping. The generalized estimating equation (GEE) analysis of SAI pretest scores indicated that neither the main effect of content theme (Wald *χ*^2^ = 0.118, *p* = 0.731 > 0.05) nor the main effect of audio reading mode (Wald *χ*^2^ = 0.584, *p* = 0.445 > 0.05) was statistically significant. Specifically, for Universal Emotional Content (Theme A), the pretest scores of the Empathic Human Voice Group (HG), Self-Reading Group (SG), and AI-Synthesized Group (AG) were 56.43 ± 11.85, 52.80 ± 12.43, and 58.85 ± 11.99, respectively (*F* = 2.536, *p* = 0.083 > 0.05); for Targeted Content (Theme B), the pretest scores of the three groups were 55.72 ± 13.49, 54.40 ± 12.77, and 56.30 ± 12.10, respectively (*F* = 0.232, *p* = 0.793 > 0.05). No statistically significant differences were observed between the two themes within each group (*p* > 0.05 for all).

Similarly, the GEE analysis of TAI pretest scores revealed that the main effect of content theme (Wald *χ*^2^ = 1.901, *p* = 0.168 > 0.05) and the main effect of audio reading mode (Wald *χ*^2^ = 0.302, *p* = 0.583 > 0.05) were not significant. Inter-group comparisons of pretest scores under both Theme A and Theme B showed no significant differences (*p* > 0.05 for all), and intra-group differences between the two themes also failed to reach statistical significance (*p* > 0.05 for all). These results indicated that the baseline emotional levels of the three groups of participants were balanced, laying a reliable foundation for the comparison of subsequent intervention effects.

#### 4.6.2 Analysis of main

Effects of Audio Reading Mode and Content ThemeAfter the intervention, both audio reading mode and content theme exerted significant impacts on college students’ anxiety levels, with their main effects being statistically significant. The GEE analysis of post-intervention SAI scores showed that the main effect of content theme (Wald *χ*^2^ = 19.469, *p* < 0.001) and the main effect of audio reading mode (Wald *χ*^2^ = 15.752, *p* < 0.001) were both significant. Correspondingly, the GEE analysis of post-intervention TAI scores also demonstrated that the main effect of content theme (Wald *χ*^2^ = 16.252, *p* < 0.001) and the main effect of audio reading mode (Wald *χ*^2^ = 15.729, *p* < 0.001) were statistically significant. These findings suggested that different audio reading modes and content themes had substantial differences in the regulation of anxiety emotions.

From the perspective of inter-group comparisons of audio reading modes, scores of both state anxiety and trait anxiety in the AG were significantly higher than those in the HG, while no significant differences were found between the SG and the other two groups. For state anxiety, inter-group differences under Theme A were significant (*F* = 4.090, *p* = 0.019 < 0.05), with the score of the AG (53.53 ± 8.83) being significantly higher than that of the HG (47.15 ± 11.03); inter-group differences under Theme B were also significant (*F* = 3.650, *p* = 0.029 < 0.05), and the score of the AG (48.00 ± 10.70) was significantly higher than that of the HG (42.00 ± 9.78). For trait anxiety, inter-group differences under Theme A were significant (*F* = 3.248, *p* = 0.042 < 0.05), with the score of the AG (49.65 ± 11.04) being significantly higher than that of the HG (43.35 ± 11.09); inter-group differences under Theme B were more significant (*F* = 4.880, *p* = 0.009 < 0.01), and the score of the AG (44.35 ± 9.32) was significantly higher than that of the HG (38.30 ± 7.99).

From the perspective of intra-group comparisons of content themes, under all three audio reading modes, Targeted Content (Theme B) had a significantly better anxiety-alleviating effect than Universal Emotional Content (Theme A). For state anxiety, the scores of Theme A in the HG (*t* = 2.209, *p* = 0.030 < 0.05), SG (*t* = 2.849, *p* = 0.006 < 0.01), and AG (*t* = 2.518, *p* = 0.014 < 0.05) were all significantly higher than those of Theme B. For trait anxiety, the scores of Theme A in the three groups were also significantly higher than those of Theme B, including the HG (*t* = 2.336, *p* = 0.022 < 0.05), SG (*t* = 2.243, *p* = 0.028 < 0.05), and AG (*t* = 2.320, *p* = 0.023 < 0.05). These results indicated that targeted content focusing on college students’ personal issues was more effective in reducing anxiety levels.

The experimental results showed that both audio reading mode and content theme had significant main effects on the regulation of college students’ anxiety emotions, without significant interactive interference (Lu et al., 2012). In terms of audio reading mode, empathic human voice reading had the optimal anxiety-alleviating effect, AI-synthesized reading had a relatively weaker effect, and self-reading was in between but showed no significant differences from the other two modes. In terms of content theme, targeted content generally had a better intervention effect than universal emotional content; regardless of the reading mode, content focusing on college students’ core concerns such as academic pressure and interpersonal growth could more effectively reduce anxiety levels. These findings verified the core hypothesis of the experiment—that empathetic transmission by professional human voices and targeted content related to individuals play a key role in emotional healing—providing empirical support for the design and optimization of subsequent mental health intervention programs (Vogl, 2013).

#### 4.6.3 Practical significance

The observed mean reductions (5–6 points on STAI, 95% CIs excluding zero) and medium effect sizes (*d* ≥ 0.47) indicate clinically and educationally meaningful improvements. For example, replacing AI-synthesized voice with empathic human voice yields an additional 6.38-point reduction in state anxiety (95% CI: 1.45–11.31), a difference sufficient to move a student from mild-to-moderate anxiety toward normal range. Self-reading provides a cost-effective alternative with moderate effects.

## 5 Discussion

The results of this study indicated a significant main effect of the audio reading mode on anxiety reduction. Specifically, participants in the Empathic Human Voice Group (HG) demonstrated a more pronounced decrease in anxiety scores compared to the AI-Synthesized Group (AG). The Self-Reading Group (SG) occupied an intermediate position, showing no statistically significant difference from either the HG or the AG. These findings provide empirical support for the first hypothesis (H1) of this study, which anticipated that the reading mode would significantly influence the intervention’s efficacy.

The observed advantage of the HG suggests that the para-linguistic features employed by professional broadcasters—such as nuanced intonational variations and deliberate rhythmic pausing—may function as potent emotional signals. From the perspective of Emotional Contagion Theory, these vocal cues likely facilitate a sense of “supportive presence,” potentially activating the listener’s emotional resonance more effectively than the relatively monotonic delivery of current AI engines. However, the lack of a significant difference between the HG and SG, as well as between the SG and AG, suggests that the “cognitive distractor” function of active reading also contributes to emotional regulation, as posited by Cognitive Resource Theory. These interpretations remain preliminary, and while the data align with the core propositions of emotional contagion and interpersonal empathy theories (Bithell, 2014), the relative efficacy of these modes may be contingent upon individual differences in cognitive load or emotional sensitivity.

Regarding the observed decline in Trait Anxiety (TA) scores, it is essential to distinguish between the structural stability of personality traits and their measurable plasticity under targeted interventions. While Trait Anxiety is traditionally conceptualized as a stable disposition, contemporary psychological research suggests that it can exhibit significant fluctuations when core cognitive distortions are addressed. Saviola et al. (2020) demonstrated that intensive psychological stimuli can down-regulate the baseline perception of environmental threats, leading to a reduction in trait-like anxiety assessments. In this study, the synergistic effect of Empathic Human Voice and Targeted Content likely provided a robust cognitive buffer for the participants. By offering specific coping strategies for academic and interpersonal stressors, the intervention facilitated a shift in the participants’ generalized appraisal of their stress environment. Consequently, the reduction in TA scores reflects an improvement in the individuals’ overall anxiety disposition during the experimental period rather than a permanent alteration of their fundamental personality structure.

The relatively weak effect of the AG may be attributed to the limitations of its emotional transmission: despite parameter adjustments to match surface features such as speech rate and volume with the HG, AI-generated speech still struggles to simulate the subtle prosodic variations and emotional logic inherent in human empathic expression. This results in lower perceived empathy and sound comfort among participants, consistent with the “mechanicalness” dilemma of AI technology in emotional expression documented in existing studies Salselas et al. (2021). Although the SG did not demonstrate a significantly superior effect compared to the AG, it achieved a certain degree of anxiety reduction through active cognitive engagement (Barak and Grohol, 2011). This finding corroborates the important role of “active cognitive participation” in emotional regulation—vocal expression and text interpretation during reading occupy cognitive resources, effectively interrupting negative rumination while enhancing participants’ sense of self-efficacy.

A core innovation of this study lies in its adoption of a 3 × 2 mixed experimental design to systematically isolate and examine the main effects of two key variables: audio reading mode and content theme (Trevarthen and Malloch, 2000). This fills a research gap in the field. In contrast to previous studies that either solely verified a single sound format or generally compared media differences, the present study features a more refined design and more actionable conclusions (Heather, 2007).

Specifically, building on prior research supporting the effectiveness of audio-based interventions, the results of this study deepen our understanding of “which type of sound is more effective.” Beyond the macro conclusion that “audio outperforms text,” it precisely identifies the micro-level difference that “empathic human voice is superior to AI-synthesized voice,” providing new empirical support for the emotional contagion theory and interpersonal empathy theory (Goldsby and Goldsby, 2020; Goldman, 2022). Meanwhile, the study’s emphasis on the targeting of content themes addresses the limitation of insufficient specificity in intervention content in previous research, promoting a shift from “generalized application” to “precision-based implementation” in audio-based interventions. Additionally, by simultaneously examining changes in both state anxiety and trait anxiety, the study not only verifies the immediate effects of the intervention but also explores its potential impacts on long-term anxiety traits, expanding the evaluation dimensions of emotional intervention efficacy (Durán et al., 2021).

Despite the significant findings, several limitations of this study should be acknowledged to ensure a balanced interpretation of the results.

First, this study employed convenience sampling, recruiting participants from a single university. This may limit the generalizability of the findings to the broader college student population or individuals from different socio-cultural backgrounds. Future studies should utilize stratified random sampling across multiple institutions to enhance ecological validity.

Second, the current experiment focused on the immediate healing effects of audio intervention. Due to the lack of longitudinal follow-up, the durability of these anxiety-reduction effects remains unknown. It is unclear whether the observed improvements persist beyond the laboratory setting or if they require repeated exposure to maintain efficacy.

Third, potential demand characteristics cannot be entirely ruled out. Since participants were aware that the study involved emotional healing, their self-reported anxiety scores might have been influenced by their expectations of the intervention’s benefits. Future research could incorporate physiological measures (e.g., heart rate variability or cortisol levels) to provide more objective evidence of emotional regulation.

Finally, the ecological validity of the laboratory setting is limited. While a controlled environment ensures internal validity, it does not fully replicate the complex and noisy real-life situations where students typically consume audio content. Further field studies are needed to investigate how these audio modes perform in daily-life scenarios.

## 6 Conclusion

Within the scope and limitations of the present experimental design—a single-session intervention with 240 Chinese college students using the STAI as the outcome measure—the following conclusions can be drawn. Among the three audio reading modes compared, empathic human voice reading demonstrated the optimal anxiety-alleviating effect, followed by self-reading, while AI-synthesized voice reading showed a relatively weaker effect. Specifically, the empathic human voice group had significantly lower post-intervention state and trait anxiety scores than the AI-synthesized group, with moderate effect sizes (Cohen’s *d* = 0.48–0.52). The self-reading group fell in between and did not differ significantly from either of the other two groups. These findings suggest that, under the present experimental conditions, the quality of emotional transmission—particularly the para-linguistic features of a professional human voice—is a key factor influencing the efficacy of audio-based anxiety reduction. Regarding content design, targeted content focusing on core concerns of college students (e.g., academic pressure, interpersonal growth, and self-identity) produced significantly greater anxiety reduction than universal emotional content across all three reading modes, with mean reductions ranging from 5.03 to 6.17 points on the STAI and medium effect sizes (Cohen’s *d* = 0.47–0.64). No significant interaction effect was found between audio reading mode and content theme, suggesting that the benefits of targeted content and empathic human voice are additive rather than synergistic under the specific conditions tested.

This study provides empirical support for the emotional contagion theory and interpersonal empathy theory by demonstrating that empathic human voice reading, which simulates real interpersonal emotional interactions, can more effectively activate emotional regulation mechanisms (Snow, 2011). The relatively weaker effect of AI-synthesized speech, likely due to limitations in emotional transmission, points to an optimization direction for affective computing: future AI emotional speech should move beyond simulating surface parameters (e.g., speech rate, volume) to incorporate prosodic features and emotional logic inherent in human empathic expression (Gaynor, 2002). The effective anxiety reduction observed in the self-reading mode corroborates the role of active cognitive participation in emotional regulation, consistent with cognitive-behavioral therapy principles (Hao et al., 2023; Gurvendra et al., 2015). The process of active vocalization and text interpretation appears to occupy cognitive resources and interrupt negative rumination, at least in the short term, suggesting that even simple active behaviors may contribute to emotional improvement and enriching the theoretical understanding of self-regulated emotional strategies (MitRahina and Costigan-Kerns, 2015).

Given the controlled nature of this study, practical recommendations should be offered with caution. For student populations similar to those in this study (Chinese college students without severe mental illness, in a laboratory setting), empathic human voice reading could be explored as a potential component in structured campus interventions, particularly for students reporting noticeable emotional distress, though its effectiveness in real-world counseling settings and over longer durations requires further validation (Goldsby et al., 2022; Beaulieu and Perez-Martinez, 2018). Self-reading, which requires no external audio resources, may serve as a convenient and cost-effective strategy for daily emotional self-regulation; it could be tentatively integrated into dormitory or classroom activities, but its sustained effects and acceptability outside the lab need investigation (Gurav et al., 2022; Crowe and Scovel, 1996). AI-synthesized reading, despite its relatively weaker effect in this study, offers advantages of low cost, replicability, and on-demand accessibility; it could be developed as a supplementary resource on campus mental health platforms to address immediate, low-intensity needs, while recognizing that its emotional transmission quality currently lags behind human voice (Magnan and Ecalle, 2006; Gurav et al., 2022). Regarding content design, the superiority of targeted content suggests that mental health materials aligned with students’ specific concerns (academic pressure, interpersonal growth, identity) may be more engaging and effective than universal emotional content; a stepped content system combining targeted and universal themes may be worth piloting, but its comparative effectiveness in real-world settings remains to be tested (Goldman and Goldman, 2017; Aung and Lee, 2004; Meneses, 2025).

The conclusions of this study are bounded by several limitations that preclude overgeneralization. The sample consisted of 240 Chinese college students recruited via convenience sampling, and findings may not generalize to other age groups, cultural contexts, or clinical populations. The intervention was delivered in a single session under controlled laboratory conditions; long-term effects and effects of repeated exposure are unknown, and real-world applicability requires further ecological validation. Reliance on self-report STAI, with no physiological or behavioral measures, is another limitation. Additionally, the self-reading group was not blinded, and the AI voice was generated by a specific engine (iFlytek); results may vary with different AI systems or reading materials. Future studies should employ longitudinal designs, diverse samples, real-world settings, and additional outcome measures to test the robustness, generalizability, and sustainability of the observed effects.

By adopting a 3 × 2 mixed experimental design, this study systematically isolates the main effects of audio reading mode and content theme, clearly revealing their independent and combined impacts on college students’ anxiety, thereby filling a research gap in the systematic comparison of different audio intervention modes and content themes (Weis et al., 1983). The simultaneous examination of changes in both state anxiety and trait anxiety verifies not only the immediate effects of the intervention but also explores its potential impacts on long-term anxiety traits, expanding the evaluation dimensions of emotional intervention efficacy (Loviasyuni and Bhuana, 2023). In summary, within the limits of the present experimental design, this study provides empirical evidence that both audio reading mode and content theme independently influence anxiety reduction in college students. Empathic human voice reading and targeted content each demonstrate measurable advantages over their counterparts, while self-reading offers a viable low-resource alternative. These findings contribute to the understanding of audio-based emotional interventions and offer preliminary, cautiously framed guidance for the development of tiered mental health resources on university campuses. Further research is essential before any broad implementation can be recommended.

## Data Availability

The original contributions presented in the study are included in the article/supplementary material, further inquiries can be directed to the corresponding author.

## References

[ref1] Aung S. K. H. Lee M. H. M. (2004). Music, sounds, medicine, and meditation: an integrative approach to the healing arts. Altern. Complement. Ther. 10, 266–270. doi: 10.1089/act.2004.10.266

[ref2] Barak A. Grohol J. M. (2011). Current and future trends in internet-supported mental health interventions. J. Technol. Hum. Serv. 29, 155–196. doi: 10.1080/15228835.2011.616939

[ref3] Bastidas Altamirán A M Ibarra Velasco M D Anxiety Reduction through the Use of Audiovisual Aids: Sixth Grade Students’ Perceptions at Marco Fidel Suarez High School Pasto, Colombia (2022).

[ref4] Beaulieu J. Perez-Martinez D. (2018). “Sound healing, theory, and practice,” in Nutrition and Integrative Medicine, ed, Aruna Bakhru (Boca Raton, Florida, USA: CRC Press), 449–469.

[ref5] Bithell C. (2014). A Different Voice, A Different Song: Reclaiming Community through the Natural Voice and World song. New York: Oxford University Press.

[ref6] Bowling A. (2005). Mode of questionnaire administration can have serious effects on data quality. J. Public Health 27, 281–291. doi: 10.1093/pubmed/fdi031, 15870099

[ref7] Braud W. (2003). Distant Mental Influence: Its Contributions to Science, Healing, and Human Interactions. Hampton Roads Publishing.

[ref8] Brown W Book-Smart: The Effect of Different Modalities of Reading Fiction on Emotional Intelligence (2025) London: Austin Macauley Publishers.

[ref005] Cohen J. (2013). Statistical Power Analysis for the Behavioral Sciences[M]. New York, NY: routledge.

[ref10] Crowe B. J. Scovel M. (1996). An overview of sound healing practices: implications for the profession of music therapy. Music. Ther. Perspect. 14, 21–29. doi: 10.1093/mtp/14.1.21

[ref11] Durán L. Almeida A. M. Figueiredo-Braga M. (2021). Digital audiovisual contents for literacy in depression: a pilot study with university students. Procedia Comp. Sci. 181, 239–246.

[ref001] Eftimova S. (2022). ONLINE BIBLIOTHERAPY PUBLICATIONS IN BULGARIA: a TRAINING ELEMENT IN THE PERIOD OF GLOBAL PANDEMIC[C]//ICERI2022 proceedings. IATED, 2816–2820. doi: 10.21427/9w9z-7z92

[ref12] Gaynor M. L. (2002). The Healing power of Sound: Recovery from life-Threatening Illness using Sound, Voice, and music. New York: Shambhala Publications.

[ref13] Goldman J. (2022). Healing Sounds: The Power of Harmonics. New York: Simon and Schuster.

[ref14] Goldman J. Goldman A. (2017). The Humming Effect: Sound Healing for Health and Happiness. New York: Simon and Schuster.

[ref15] Goldsby T. L. Goldsby M. E. (2020). Eastern integrative medicine and ancient sound healing treatments for stress: recent research advances. Int. J. Res. Med. Sci. 19:24. doi: 10.18203/2320-6012.ijrms20174563, 33488307 PMC7819493

[ref16] Goldsby T. L. Goldsby M. E. McWalters M. Mills P. J. (2022). Sound healing: mood, emotional, and spiritual well-being interrelationships. Religion 13:123. doi: 10.3390/rel13020123

[ref17] Gurav K. M. Kulkarni N. Shetty V. Vinay V. Borade P. Ghadge S. . (2022). Effectiveness of audio and audio-visual distraction aids for management of pain and anxiety in children and adults undergoing dental treatment-a systematic review and meta-analysis. J. Clin. Pediatr. Dent. 46, 86–106. doi: 10.17796/1053-4625-46.2.2, 35533223

[ref18] Gurvendra G lal Gurvendra A Kulkarni J Effect of Sound Healing Meditation on Increased Anxiety & Stress in Women (2015). India: Saraswat Publishers Private Limited Delhi.

[ref19] Hao T. Pang J. Liu Q. Xin P. (2023). A systematic review and network meta-analysis of virtual reality, audiovisuals and music interventions for reducing dental anxiety related to tooth extraction. BMC Oral Health 23:684. doi: 10.1186/s12903-023-03407-y, 37735362 PMC10515077

[ref003] Hatfield E. Cacioppo J. T. Rapson R. L. (1993). Emotional contagion. Curr. Dir. Psychol. Sci. 2, 96–100. doi: 10.1111/1467-8721.ep10770953

[ref20] Heather S. (2007). What is sound healing. Int. J. Heal. Caring 7, 1–11.

[ref21] Hill C. E. Helms J. E. Tichenor V. Effects of Therapist Response Modes in Brief Psychotherapy (ed.) Hill W.F., Allyn Bacon Boston, MA, USA: Bastidas Altamirán & Ibarra (2001).

[ref22] Jain S. McKusick E. Ciccone L. Sprengel M. Ritenbaugh C. (2023). Sound healing reduces generalized anxiety during the pandemic: a feasibility study. Complement. Ther. Med. 74:102947. doi: 10.1016/j.ctim.2023.102947, 37023932

[ref24] Kim N. Y. (2021). E-book, audio-book, or E-audio-book: the effects of multiple modalities on EFL comprehension. Engl. Teach. 76, 33–52. doi: 10.15858/engtea.76.4.202112.33

[ref25] Kujala T. Karma K. Ceponiene R. Belitz S. Turkkila P. Tervaniemi M. . (2001). Plastic neural changes and reading improvement caused by audiovisual training in reading-impaired children. Proc. Natl. Acad. Sci. 98, 10509–10514. doi: 10.1073/pnas.181589198, 11517333 PMC56991

[ref26] Kulakaç N. Ustuner Top F. (2025). The Effect of Audio-Visual Methods on Preoperative Anxiety in Children: A Systematic Review and Meta-Analysis of Randomized Controlled Trials[C]//Child & Youth Care Forum. New York: Springer.

[ref008] Lazarus R. Folkman S. (1985). Stress and coping[J]. N. Y. 18, 34–42.

[ref27] Lee S. P. Lee S. D. Liao Y. L. Wang A. C. (2015). Effects of audio-visual aids on foreign language test anxiety, reading and listening comprehension, and retention in EFL learners. Percept. Mot. Skills 120, 576–590. doi: 10.2466/24.PMS.120v14x2, 25914939

[ref28] Liu Y. (2024). A study of the effects of four reading styles on college students’ mental health and quality of life based on positive psychology-a first-of-its-kind study. PLoS One 19:e0308475. doi: 10.1371/journal.pone.0308475, 39196887 PMC11355533

[ref29] Livingstone S. R. Russo F. A. (2018). The Ryerson audio-visual database of emotional speech and song (RAVDESS): a dynamic, multimodal set of facial and vocal expressions in north American English. PLoS One 13:e0196391. doi: 10.1371/journal.pone.0196391, 29768426 PMC5955500

[ref0010] Li W. Qian M. Y. (1995). Revision of the state-trait anxiety inventory with Chinese sample. Acta Sci. Nat. Univ. Pekin. 31, 108–112.

[ref30] Loviasyuni N. E. Bhuana G. P. (2023). Audio-visual as media in reading: students’ responses and challenges. J. Eng. Lang. Teach. Learn. Linguist. Literat. 11, 607–615. doi: 10.24256/ideas.v11i1.4003

[ref31] Lu H Frauendorfer D Rabbi M Stresssense: detecting stress in unconstrained acoustic environments using smartphones Proceedings of the 2012 ACM conference on ubiquitous computing (2012) 351–360

[ref32] Magnan A. Ecalle J. (2006). Audio-visual training in children with reading disabilities. Comput. Educ. 46, 407–425. doi: 10.1016/j.compedu.2004.08.008

[ref33] Meneses E. Harmonic Healing: The Influence of Sound Therapy on Cultivating Forgiveness (2025). Oxfordshire, UK: Routledge Abingdon.

[ref34] MitRahina K Costigan-Kerns L Healing Potentials of Sound and Music (2015) New York: Sound Healing Research Foundation.

[ref35] Nettifee M. (2020). Voice as Embodied sense: Rethinking Voice and Language in Trauma Healing. Long Beach, California: Pacifica Graduate Institute.

[ref0012] O’Connell N. S. Dai L. Jiang Y. Speiser J. L. Ward R. Wei W. . (2017). Methods for analysis of pre-post data in clinical research: a comparison of five common methods. Journal of Biometrics & Biostatistics 8, 1–8. 30555734 10.4172/2155-6180.1000334 PMC6290914

[ref37] Oktay S. Söğütlüler T. Avcil S. Akçanal Şeker C. Öner H. Öztürk H. . (2025). Using audiovisual intervention to reduce anxiety and improve image quality in pediatric magnetic resonance imaging. Pediatr. Radiol. 55, 1582–1590. doi: 10.1007/s00247-025-06276-5, 40471260 PMC12321924

[ref38] Pennebaker J. W. (2012). Opening Up: The Healing power of Expressing Emotions. New York: Guilford Press.

[ref39] Potamianos G. Neti C. Luettin J. . (2004). Audio-visual automatic speech recognition: an overview. Issues Vis. Audio Visual Speech Process. 22:23.

[ref40] Rogowsky B. A. Calhoun B. M. Tallal P. (2016). Does modality matter? The effects of reading, listening, and dual modality on comprehension. SAGE Open 6:2158244016669550. doi: 10.1177/2158244016669550

[ref002] Salselas I. Penha R. Bernardes G. (2021). Sound design inducing attention in the context of audiovisual immersive environments. Pers. Ubiquit. Comput. 25, 737–748. doi: 10.1007/s00779-020-01386-3

[ref007] Saviola F. Pappaianni E. Monti A. Grecucci A. Jovicich J. De Pisapia N. (2020). Trait and state anxiety are mapped differently in the human brain. Sci. Rep. 10:11112. doi: 10.1038/s41598-020-68008-z 32632158 PMC7338355

[ref0011] Senn S. J. (2006). Change from baseline and analysis of covariance revisited. Stat. Med. 25, 4334–4344. doi: 10.1002/sim.2682 16921578

[ref41] Snow S. (2011). Healing through Sound: An Exploration of a vocal Sound Healing Method in Great Britain. New York: Concordia University.

[ref9001] Speilberger C. D. Gorsuch R. L. Lushene R. (1983). Manual for the State-Trait Anxiety Inventory[J]. Palo Alto, CA: Consulting Psychologists.

[ref004] Sweller J. (1988). Cognitive load during problem solving: effects on learning. Cogn. Sci. 12, 257–285. doi: 10.1207/s15516709cog1202_4

[ref42] Taylor S. E. Thompson S. C. (1982). Stalking the elusive" vividness" effect. Psychol. Rev. 89:155. doi: 10.1037/0033-295x.89.2.155

[ref44] Trevarthen C. Malloch S. N. (2000). The dance of wellbeing: defining the musical therapeutic effect. Nor. Tidsskr. Musikkter. 9, 3–17. doi: 10.1080/08098130009477996

[ref45] Tsai S. L. (2004). Audio-visual relaxation training for anxiety, sleep, and relaxation among Chinese adults with cardiac disease. Res. Nurs. Health 27, 458–468. doi: 10.1002/nur.20039, 15514963

[ref46] Vogl S. (2013). Telephone versus face-to-face interviews: mode effect on semistructured interviews with children. Sociol. Methodol. 43, 133–177.

[ref47] Wang M. Sun H. (2024). An analysis of artistic expressions and healing elements in the morphological design of picture books. Cult. Int. J. Philos. Cult. Axiol. 21:213–234.

[ref48] Weis O. F. Abrahamson W.G. McCrea K. D. Satler J.F. Zangerl A.R. Anderson S.S. . (1983). Reduction of anxiety and postoperative analgesic requirements by audiovisual instruction. Lancet 321, 43–44. 10.1016/s0140-6736(83)91574-x 6184582

[ref49] Yang Z. Zhao X. Zhu L. Xia Y. Ma Y. Wu J. . (2023). Research on the healing potential of urban parks from the perspective of audio-visual integration: a case study of five urban parks in Chengdu. Land 12:1317. doi: 10.3390/land12071317

[ref006] Zheng X. Fang F. Nong W. . (2021). Development and validation of a model to estimate the risk of acute ischemic stroke in geriatric patients with primary hypertension[J]. BMC Geriatr. 21:458. doi: 10.1186/s12877-021-02392-7 34372766 PMC8353783

